# Exteriorization of Buried Port to Salvage Infected Tissue Expander

**Published:** 2009-09-08

**Authors:** Ahmed Elshahat

**Affiliations:** Ain Shams University, 38 Elshaheed Said Afify Ali St, Ard Elgolf, Nasr City, 11371 Cairo, Egypt

## Abstract

**Objective:** Since removal of an infected tissue expander is very disappointing to both the surgeon and the patient, every effort is directed toward its salvage. This study evaluates a new method to salvage infected tissue expanders. **Method:** Of 66 tissue expanders applied at different sites in the body, 12 developed infection. Salvage was carried out by exteriorizing the buried port, followed by irrigation through the pocket of the tube that connects the port to the expander. **Result:** Salvage was successful in 9 of the infected tissue expanders and failed in 3 cases. The ports were not dependent in these 3 cases. **Conclusion:** Exteriorization of dependent ports allows adequate drainage, good access for irrigation, and completion of expansion.

Radovan^1^ in 1982 and Austad and Rose^2^ in the same year introduced the use of tissue expanders to manage difficult reconstructive situations. While most surgeons adopted the buried port technique, some advocated external filling ports.^3^–^9^

Infection of an expander is a devastating complication and traditional surgical principles dictate removal of the device.^10^ Salvage has become an increasingly acceptable alternative to removal.^10^–^13^

Yii and Khoo^10^ and Kendrick and Chase^12^ advocated removing the infected expander, followed by irrigation, and then reinsertion of a new expander. On the other hand, Sugden et al^11^ and Kajikawa et al^13^ irrigated the infected periexpander pocket without removal of the expander.

While Sugden et al^11^ entered the pocket through the skin over the integral port and used needles for irrigation and aspiration, Kajikawa et al^13^ reached the periexpander pocket through incision on the expander site and inserted a drainage tube into the pocket for irrigation and aspiration.

This study evaluates the efficacy of exteriorizing the buried port to salvage infected tissue expanders.

## PATIENT AND METHODS

A single surgeon (the author) inserted 66 tissue expanders at different location in the body. Twelve expanders developed infection. Location of expanders, number at each location, and the number of infected expanders at each location are shown in Table 1.

All expanders were used to reconstruct postburn and posttraumatic deformities (alopecia, scarring, or contractures) except 9. One was used on the scalp to enable reconstruction of the scalp before excision of a cirsoid aneurysm. Three were used in the abdomen to facilitate full harvesting of full-thickness skin graft. Five were used for breast reconstruction after mastectomy. All expanders had their ports buried. Infection was noticed after more than 30% of expansion was reached in 8 cases and after more than 80% of expansion in 4 cases.

The earliest sign of detection of infection was collection of fluid over the port, which exuded following withdrawal of the injecting needle. This sign appeared early before signs of inflammation. Management of infected expanders included exteriorization of buried ports under local anesthesia, which allowed drainage of collected purulent fluid. This was followed by irrigation of the pocket with gentamicin (Garamycin) along the connecting stem. Care was taken to avoid damaging the ports during exteriorization.

Full expansion (100%) was reached in all salvaged expanders. Systemic antibiotics were administered for 2 weeks after exteriorization to be sure of complete resolution of infection. There was no expansion during this period. Antibiotic regimen was not continued.

After completion of expansion, a 2-week waiting period was used before the removal of expanders and advancement of the flaps. In cases of breast reconstruction, overexpansion was performed and a 3-month waiting period was used before replacing the expander with a permanent implant.

## RESULTS

Exteriorization of the buried ports in cases of infected expanders allowed drainage of the purulent fluid and greatly improved the general condition of the patients. This technique prevented spontaneous drainage of pus from weak points in the skin covering the expanders and thus prevented exposure.

**Figure 1:**
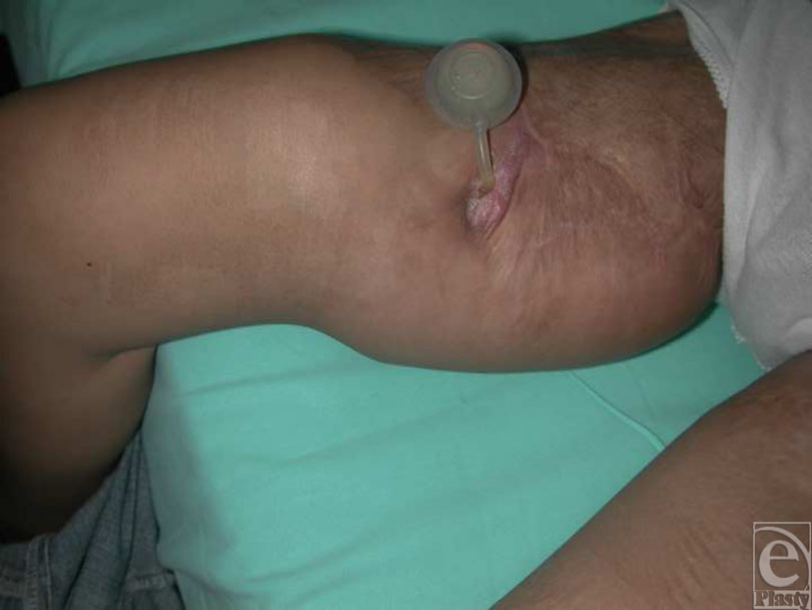
Exteriorized port of a tissue expander located at the medial aspect of the right thigh. Exteriorization salvaged the infected tissue expander and allowed completion of the expansion.

Salvage was successful in 9 of the 12 infected expanders and the expansion was completed to the planned volume (Fig 1).

The common feature in the 3 infected cases that failed to be salvaged was the position of port in the nondependent location that prevented free drainage with gravity. Failure of salvage in these cases necessitated the termination of the procedure and removal of the expanders, but still exteriorization of the ports in these 3 cases allowed the removal of expanders as elective rather than emergency surgical procedures.

Culture of drained collections showed growth of *Staphylococcus aureus* in 10 cases but no growth in 2 cases. Appropriate anti gram-positive antibiotic (amoxicillin + clavulanic acid) was prescribed.

Intraoperatively, the pocket lining was gelatinous and inflamed in the 3 nonsalvaged cases. The lining looked healthy in salvaged expanders; however, no biopsies were performed.

One of the 3 nonsalvaged expanders was used for breast reconstruction postmastectomy. The expander was removed and the patient was lost to follow up.

## DISCUSSION

Despite all precautions taken to prevent infection of tissue expanders, this is still a major complication that may necessitate removal of the appliance. Early infection results from the introduction of bacteria in the perioperative period, whereas late infection results from iatrogenic introduction of bacteria during the course of expansion.^14^

Keskin et al,^9^ who used external ports, mentioned that during the early stages of expansion, it is not rare to see drainage from the site where the filling tube penetrates skin and stressed that this should not be mistaken for infection. They add that as daily expansion proceeds, any remaining seroma or hematoma will drain from the entrance site of the filling tube.

This possibility of drainage through the entrance site of filling tubes encouraged me to think that periexpander collection might drain by exteriorizing the buried ports.

It is very important during the planning stage to put the port in dependent areas that allows free drainage once the port has been exteriorized. The common factor in the 3 infected expanders that failed to be salvaged was that the port was located in nondependent locations that prevented free drainage.

It is essential for success of this technique, which allows drainage of infection by exteriorizing the port, to detect infection and purulent collection as early as possible. Early detection while the collection is seropurulent is more likely to be successful than those cases of late detection when the collection becomes unmistakably purulent.

Salvage of the infected expanders by exteriorizing the ports is a very efficient technique that allows free drainage of purulent collection and gives access for irrigation.

## Figures and Tables

**Table 1:** Location of application of expanders, number of total expanders at each location, and the number of infected expanders at each location

Location	Number of total expanders	Number of infected expanders
Scalp	20	1
Neck	12	2
Back	6	1
Breast	5	1
Abdomen	9	1
Arm and forearm	6	2
Thighs	8	4
